# Abiotic Stresses Shift Belowground *Populus*-Associated Bacteria Toward a Core Stress Microbiome

**DOI:** 10.1128/mSystems.00070-17

**Published:** 2018-01-23

**Authors:** Collin M. Timm, Kelsey R. Carter, Alyssa A. Carrell, Se-Ran Jun, Sara S. Jawdy, Jessica M. Vélez, Lee E. Gunter, Zamin Yang, Intawat Nookaew, Nancy L. Engle, Tse-Yuan S. Lu, Christopher W. Schadt, Timothy J. Tschaplinski, Mitchel J. Doktycz, Gerald A. Tuskan, Dale A. Pelletier, David J. Weston

**Affiliations:** a Biosciences Division, Oak Ridge National Laboratory, Oak Ridge, Tennessee, USA; b Joint Institute for Biological Sciences, University of Tennessee, Knoxville, Tennessee, USA; c School of Forest Resources and Environmental Science, Michigan Technological University, Houghton, Michigan, USA; d University of Arkansas for Medical Sciences, Little Rock, Arkansas, USA; University of Nebraska-Lincoln

**Keywords:** drought, microbiome, poplar, shading

## Abstract

Adverse growth conditions can lead to decreased plant growth, productivity, and survival, resulting in poor yields or failure of crops and biofeedstocks. In some cases, the microbial community associated with plants has been shown to alleviate plant stress and increase plant growth under suboptimal growing conditions. A systematic understanding of how the microbial community changes under these conditions is required to understand the contribution of the microbiome to water utilization, nutrient uptake, and ultimately yield. Using a microbiome inoculation strategy, we studied how the belowground microbiome of *Populus deltoides* changes in response to diverse environmental conditions, including water limitation, light limitation (shading), and metal toxicity. While plant responses to treatments in terms of growth, photosynthesis, gene expression and metabolite profiles were varied, we identified a core set of bacterial genera that change in abundance in response to host stress. The results of this study indicate substantial structure in the plant microbiome community and identify potential drivers of the phytobiome response to stress.

**IMPORTANCE** The identification of a common “stress microbiome” indicates tightly controlled relationships between the plant host and bacterial associates and a conserved structure in bacterial communities associated with poplar trees under different growth conditions. The ability of the microbiome to buffer the plant from extreme environmental conditions coupled with the conserved stress microbiome observed in this study suggests an opportunity for future efforts aimed at predictably modulating the microbiome to optimize plant growth.

## Abstract (precis)

The identification of a common “stress microbiome” indicates tightly controlled relationships between the plant host and bacterial associates and a conserved structure in bacterial communities associated with poplar trees under different growth conditions. The ability of the microbiome to buffer the plant from extreme environmental conditions coupled with the conserved stress microbiome observed in this study suggests an opportunity for future efforts aimed at predictably modulating the microbiome to optimize plant growth.

## INTRODUCTION

The microbiome has the capacity to act as an extension of the host genotype that can respond to changes in environmental conditions and evolve rapidly (1). Changes in the host organism or environment have been shown to shift the composition of the associated microbiota in humans (2, 3), mice (4), coral (5), and plants (6). The additional functions encoded by members of the plant microbiome can modify nutrient uptake (7, 8), produce (9) or degrade (9–11) plant hormones, prime host defense pathways against pathogens (12) and pests (13), and ultimately affect both the above- and belowground growth of the host plant (14–16). In communities, effects of individual members can be additive (17), synergistic (18–20), or antagonistic (21, 22). As a complex community, the plant belowground microbiome has been shown to adapt rapidly to water limitation conditions and alleviate host stress (23, 24). This ability of individual microbes and microbial communities to modify plant growth characteristics and alleviate stress suggests an opportunity to optimize plant growth for biomass and yield, either by increasing plant growth and productivity or by modulating metabolite profiles for downstream biomass processing. Toward this goal, a thorough understanding of the plant-microbiome relationship along with the response of the microbiome to changes in the host and environment is required.

Members of the *Populus* genus, fast-growing trees that are candidate second-generation biofuel feedstocks, are an attractive system for studying such microbiome intervention strategies. Recent 16S rRNA gene profiling studies of *Populus* growing in its natural habitat have contributed to our understanding of the *Populus* root microbiome community structure (25–28). These studies have demonstrated that *Populus* roots are host to a diverse bacterial community that differs on the basis of soil, geographic location, season, and host genotype but is most strongly influenced by the ecological niche (e.g., rhizosphere versus endosphere). Rhizosphere communities demonstrate an abundance of *Acidobacteria* that were greatly diminished in the endosphere (defined as within surface-sterilized root tissues), while *Alphaproteobacteria* and *Gammaproteobacteria*, along with *Actinobacteria*, were enriched in the root endosphere. Understanding the interplay between rhizosphere and root communities is critical for tailored microbiome intervention strategies to improve plant productivity.

The production of biomass for food or energy crops will ultimately be affected by environmental conditions such as water and light availability and the presence of toxins. Climate change has led to changing precipitation regimes and more extreme weather events (29), and the ability of the microbiome to buffer against water limitation (23) provides an opportunity to mitigate the effect of these conditions in the field. Water limitation conditions have been shown to affect the microbiome by decreasing mycorrhizal colonization, ultimately decreasing nutrient acquisition by the host plant (30). Shading and cloud cover are natural limitations on light, decrease overall biomass production, and lead to structural changes in *Populus* species (31). These inhibitors have been shown to impact metabolite profiles in tomato (32) and tea (33) plants. Shading can significantly affect plant metabolite profiles and has been proposed as a method for optimizing secondary metabolite production (34). Shading, and the ultimate effect on plant photosynthesis and carbon allocation, also shifts the association of the plant with beneficial microbes in the environment (35). Finally, the presence of toxins and inorganic chemicals in the environment impacts the microbial community directly as antimicrobial compounds (36) or indirectly by either inhibiting the proliferation of other community members (37) or modifying the host exudate and chemical profile. Copper is an essential micronutrient for plants, but at high concentrations, it can inhibit plant growth in rice (38). In *Populus*, excess copper is accumulated in roots and decreases leaf chlorophyll content and photosynthesis (39, 40).

Responses to diverse stresses have been used to study the biology of bacteria (41, 42), yeast (43, 44), and plants (45–47). These studies identified core responses that were conserved across stress treatments which helped map the functions of genes and proteins in stress response. Gene expression studies show that while plants encode a wide range of mechanisms to deal with unique stresses, there is a subset of genes that are regulated in response to generic stress (45–51). For example, similar patterns of unique and core responses were observed in metabolite profiles in *Zea mays* (maize) responses to drought and heat stress (52). Similar to analysis of individual genes on host function, individual microbiome members have been shown to modulate gene expression on the basis of the presence of plant metabolites (53, 54), suggesting a response to changes in the host metabolome, and indeed, the microbiome community has been linked to changes in the chemical environment of the host in engineered lignin mutants of *Populus* (55). In this work, we aimed to determine if a core response occurs in the microbiome of plants subjected to diverse stresses. Understanding how the plant and its associated microbiome respond to changes in the environment is critical for harnessing the protective and adaptive powers of the microbiome. We hypothesized that subsets of the plant belowground microbiome community (root and rhizosphere) would mirror the host response to stress, showing both a treatment-specific response and a core stress response showing microbial abundance changes that are shared between stress treatments. Using a microbiome inoculation strategy, we studied how *Populus deltoides* and its associated belowground microbiome respond to abiotic stresses of water limitation, shading, and copper toxicity.

## RESULTS

### Stress conditions reduced plant growth and altered physiology.

**FIG 1:**
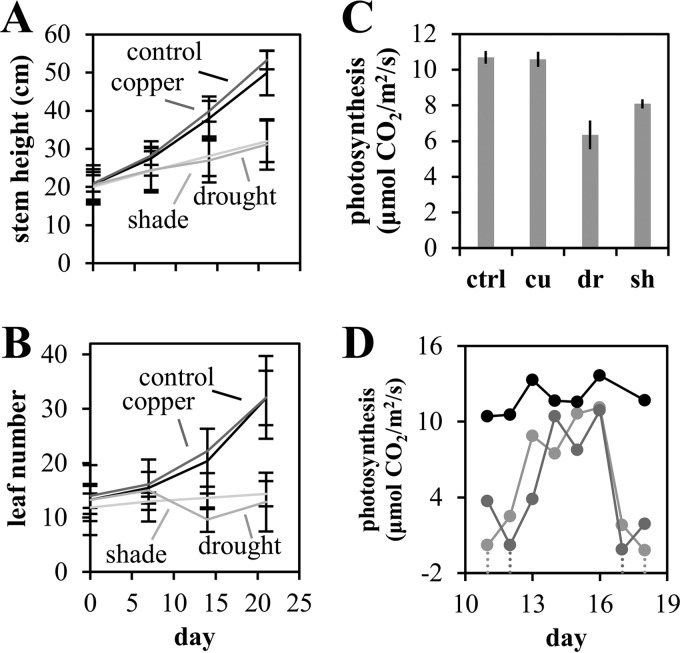
Plant growth and physiology. (A) Stem height was measured to the apical meristem. (B) Leaves longer than 2 cm were counted for leaf numbers. Error bars in panels A and B are standard errors from eight plants per condition at each point. (C) CO_2_ gas exchange rate at a PAR level of 400 µmol m^−2^ s^−1^ for control (ctrl) and copper (cu)-, drought (dr)-, and shade (sh)-treated plants. Error bars in panel C are 1 standard error of the mean (standard deviation/mean) from 10, 8, 10, and 9 (control, copper, drought, and shade, respectively) measurements across 3 days. (D) Photosynthesis rate for two drought plants (gray lines) and one control plant (black line) throughout a drought cycle. Watering events are indicated by vertical dotted lines.

Inoculation of *P. deltoides* WV94 rooted cuttings (eight plants per conditions) with the wild microbiome resulted in increased leaf mass per area (LMA; 4.70 versus 5.13 mg/cm^2^; *P* < 0.05 [Student's *t* test]) and a decrease in the Ball-Berry parameter (summarizes the relationship between stomatal conductance and net photosynthesis [0.024 versus 0.020; *P* < 0.05 by Student's *t* test]) and different microbiome communities compared to uninoculated controls (*P* < 0.001, Fig. S1; see Data Set S1 [Clustering Statistics] in the supplemental material), indicating that the plants were colonized by the natural microbiome when this microbiome inoculation method was used. Uninoculated control plants were colonized by microbes from the greenhouse environment and were included primarily to ensure that the inoculation strategy resulted in a microbiome representative of that in other *Populus* studies. After acclimation to greenhouse conditions (~2 weeks), inoculated plants were subjected to one of three environmental stressors, water limitation (cyclic drought based on individual plant responses with plants reaching drought conditions three to five times throughout the treatment period; Fig. S1), shade (80% light interception), or heavy metal toxicity (30 µM CuSO_4_ in nutrient solution). Stem height and leaf number were measured weekly during treatments (Fig. 1A and B) and were significantly reduced by the end of the treatment in shaded plants (height, *P* < 0.001; leaf number, *P* < 0.001 [Student's *t* test]) and water-limited plants (height, *P* < 0.001; leaf number, *P* < 0.001 [Student's *t* test]). In water-limited plants, the LMA was increased (5.80 versus 5.13 mg/cm^2^; *P* < 0.05 [Student's *t* test]), consistent with a decreased surface area to reduce water loss to the environment (Fig. S2). In shaded plants, the total leaf area and LMA were significantly reduced (leaf area, 600 versus 1,890 cm^2^; *P* < 0.001; LMA, 3.26 versus 5.13 mg/cm^2^; *P* < 0.001 [Student's *t* test]), consistent with similar light limitation studies with *P. deltoides* ×* P. trichocarpa* hybrids (31). Copper treatment resulted in the accumulation of copper in leaf tissues (1.39× increase; *P* < 0.05 [Student's *t* test]) (Fig. S2).

10.1128/mSystems.00070-17.1 FIG S1  Effect of natural microbiome on plant phenotype and belowground microbiome community. (A) LMA measured from three 1-cm leaf punches at harvest (Student's *t* test, *P* < 0.05, 8 plants each). (B) Transpiration rate (Student's *t* test, *P* < 0.05, 6 and 5 plants). (C) Ball-Berry parameter summarizing the relationship between stomatal conductance and net photosynthesis (Student's *t* test, *P* < 0.05). Error bars show standard errors in all of the plots. (D) PCoA1 and PCoA2 of root samples only. (E) PCoA2 and PCoA3 of the weighted UniFrac distance metric for root samples only. (F) PCoA1 and PCoA2 of rhizosphere (rhiz.) samples only. (G) PCoA2 and PCoA3 of rhizosphere samples only. Download FIG S1, TIF file, 0.2 MB. Copyright © 2018 Timm et al. 2018 Timm et al. This content is distributed under the terms of the Creative Commons Attribution 4.0 International license.

10.1128/mSystems.00070-17.2 FIG S2  Effect of natural microbiome on plant phenotype. (A) Total leaf area measured at harvest (*, *P* < 0.05 [Student's *t* test]; 8 plants each). (B) LMA measured from three 1-cm leaf punches at harvest (*P* < 0.05 [Student's *t* test]; 8 plants each). (C) Respiration rate (µmol of CO_2_ m^−2^ s^−1^). (D) Net photosynthesis (µmol of CO_2_ m^−2^ s^−1^). (E) Transpiration rate (*, *P* < 0.05 [Student's *t* test]; 6 and 5 plants). (F) Stomatal conductance to water vapor. (G) Intercellular CO_2_ concentration. (H) Quantum yield (phiCO_2_). Error bars show standard errors in all plots. Download FIG S2, TIF file, 1.7 MB. Copyright © 2018 Timm et al. 2018 Timm et al. This content is distributed under the terms of the Creative Commons Attribution 4.0 International license.

10.1128/mSystems.00070-17.5 DATA SET S1  Excel spreadsheet with supplemental data. Parts: 1, leaf metabolites measured by GC-MS presented as abundance and *P* values (Student's *t* test, four samples per treatment); 2, qRT-PCR results for gene expression including *Populus* gene, *Arabidopsis* homolog, and pathway; 3, core OTUs for root and rhizosphere including the taxonomy list of the central subnetwork in Fig. 5B; 4, statistical results for microbial community 16S amplicon cluster comparisons; 5, omics correlations for combined analysis of OTUs, metabolites, and gene expression data; 6, barcodes for 16S amplicon data analysis. Download DATA SET S1, XLSX file, 0.4 MB. Copyright © 2018 Timm et al. 2018 Timm et al. This content is distributed under the terms of the Creative Commons Attribution 4.0 International license.

Plant chlorophyll fluorescence and gas exchange parameters were measured in two sets: on days 17 and 18 of stress conditions for four plants per condition and daily on days 12 to 18 for two drought plants and two control plants to track drought cycles. Plants were randomly selected to complete measurements between 10 am and 2 pm on subsequent days. Net photosynthesis and water use were significantly reduced in water-limited plants (6.4 versus 10.7 µmol CO_2_ m^−2^ s^−1^; *P* < 0.01 [Student's *t* test]) and shade-treated plants (8.1 versus 10.7 µmol CO_2_ m^−2^ s^−1^; *P* < 0.01 [Student's *t* test]) (Fig. 1D; Fig. S2). Interestingly, we observed an increase in instantaneous water use efficiency in copper-treated plants (5.8 versus 3.5 µmol CO_2_/µmol H_2_O; *P* < 0.01 [Student's *t* test]). The CO_2_ respiration rate was significantly reduced in water-limited and shade-treated plants (water limited, 1.5 versus 2.1 µmol CO_2_ m^−2^ s^−1^, *P* < 0.01; shade treated, 1.0 versus 2.1, *P* < 0.001 [Student's *t* test]), suggesting decreased metabolic activity in the leaves of stressed plants.

### Plant transcriptional response to stress.

**FIG 2:**
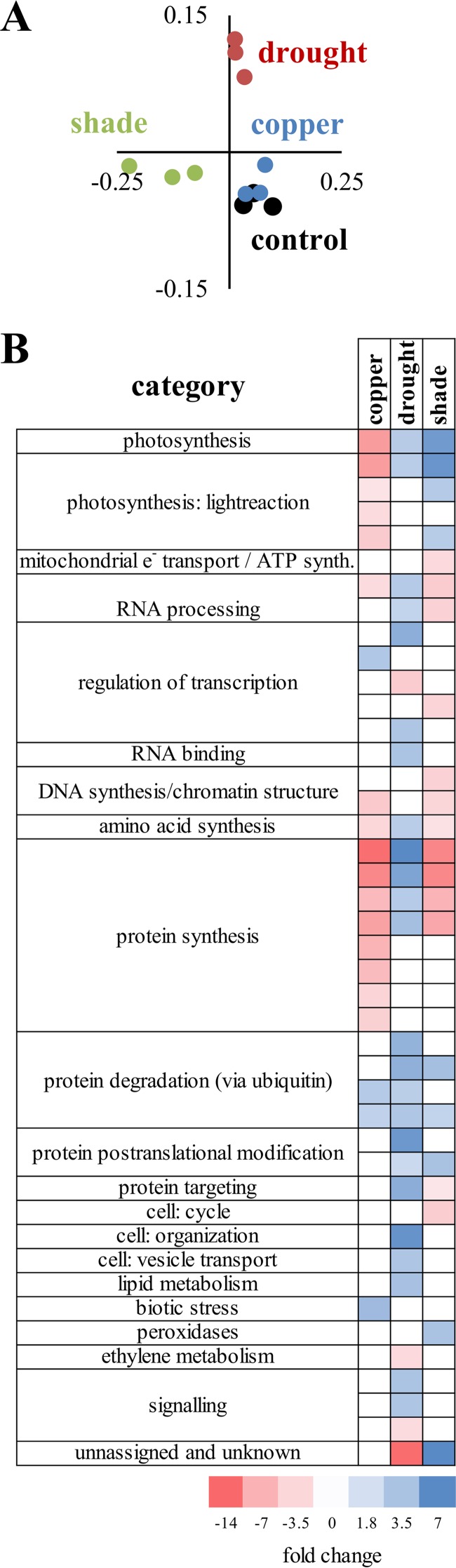
Plant transcriptional responses to treatments. Plant transcriptomes were sequenced (three per condition) and analyzed. (A) PCoA of normX expression profile. (B) PageMan analyses to determine over- and underrepresentation in treatments.

Plant tissue was collected at the end of the stress treatments and measured for transcriptional response by transcriptome sequencing (three plants per condition, excluding uninoculated controls). Transcriptional profiles clustered by treatment (Fig. 2A) and were analyzed to determine significant over- and underrepresentation of responsive groups (Fig. 2B). Genes annotated to participate in photosynthesis were downregulated relative to the control in copper-treated plants. Specifically, photosystem I (PSI) was decreased (−2.51-fold), with the gene ontology (GO) category for PSI polypeptide subunits decreased −3.05-fold. Photosystem II (PSII) was also decreased (−6.07-fold), with both LHC-II (−2.97-fold) and PSII polypeptide subunits decreased (−4.63-fold). In drought and shade treatments, photosynthesis genes were upregulated, consistent with carbon starvation. Specifically, the GO category annotated as light reaction was upregulated 2.92-fold in water-limited plants and 6.4-fold in light-limited plants, with the ATP synthase and cytochrome *b*_6_/f GO categories upregulated in light-limited plants (3.1- and 2.1-fold). Additionally, the GO category annotated as PSII genes was upregulated 4.1-fold, with the PSII polypeptide subunit group upregulated 3.0-fold. RNA, DNA, and protein metabolism were downregulated in copper- and shade-treated plants but upregulated in drought plants relative to the controls. Copper-treated plants showed downregulation relative to the control of chloroplast ribosomal proteins, consistent with the copper ion interacting with photosynthetic membranes. Drought-treated plants showed an increase in the expression of genes associated with lipid metabolism, as well as cellular organization and vesicle transport, relative to the controls (Fig. 2). The common change observed across treatments was an increase in the expression of protein degradation pathways. We also observed responses in hormone signaling, cell structure, and stress pathways. These results are supported by complementary quantitative reverse transcription (qRT)-PCR analysis of the expression of a panel of selected genes (Data Set S1). Together, these results indicate a unique response of plants to each treatment.

### Global metabolite shifts indicate a specific host response to stress.

**FIG 3:**
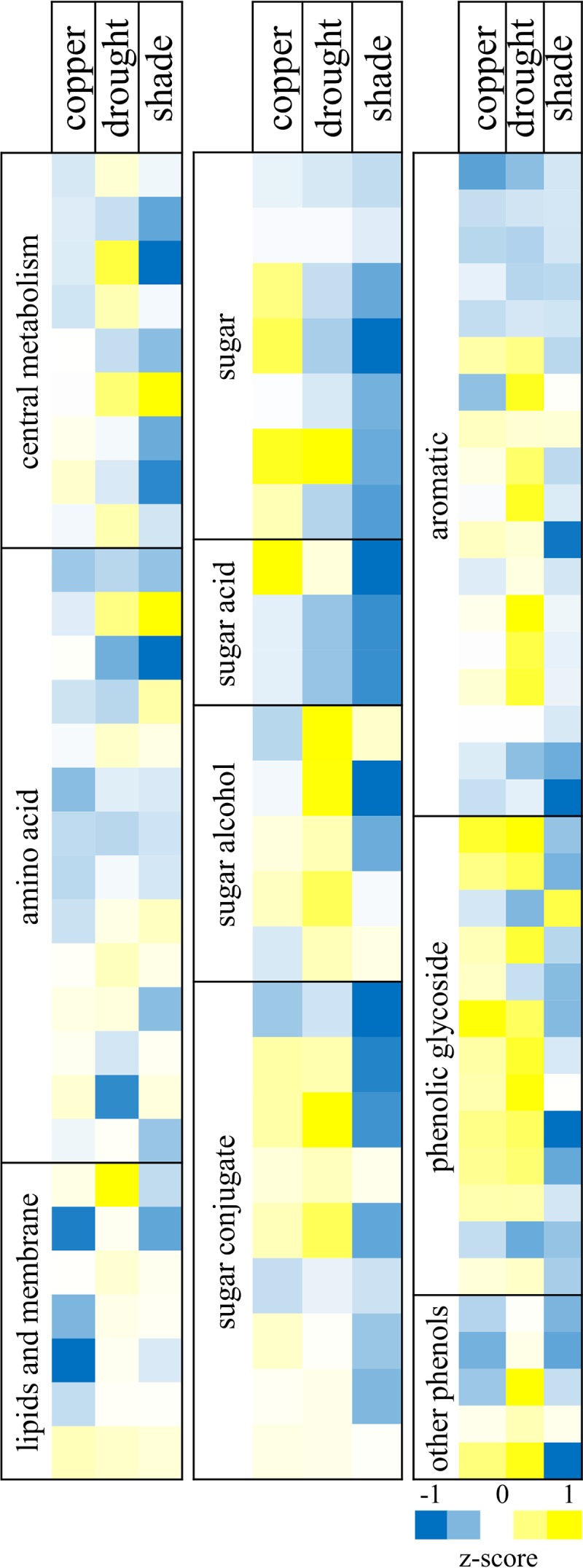
Leaf metabolite profile changes. Log_2_ expression values are shown. Blue indicates a decrease relative to the control, and yellow indicates an increase. Colors are z scaled within each compound group (i.e., amino acids, sugars, etc.).

Leaf metabolites were measured at the end of the treatments by using gas chromatography-mass spectrometry (GC-MS) for four plants per treatment. We detected 122 unique peaks and partially or fully identified 95 metabolites, which were further classified into groups on the basis of host pathways or chemical functionality (Fig. 3). Consistent with the transcriptome response, metabolites in the lipid synthesis group were upregulated in water-limited plants. In copper-treated plants, we observed high levels of aromatics and sugar conjugates, including phenolic glycosides. Central metabolites were moderately affected by water limitation and copper treatments. Though copper induced a moderate decrease in citrate, light limitation resulted in a decrease in multiple tricarboxylic acid (TCA) cycle metabolites (glycerate, succinate, methylmalonate), and significant increases in aconitate (Student's *t* test, *P* < 0.05). These changes are consistent with other studies of the TCA cycle response to light (56). Metabolites in the amino acid group showed similar response patterns between stresses and in general followed patterns predicted by transcriptome analyses. Alanine, glutamate, aspartate, and the precursor 5-oxo-proline were all decreased in response to the copper, water limitation, and shade treatments, while glycine increased. In light-limited plants, glutamate and gamma-aminobutyric acid increased while serine decreased. Lipids and membrane-associated metabolites were affected the most by copper treatment, with mono- and digalactosylglycerol decreased 1.7-fold. The aromatic group includes a distinct set of metabolites (including gallate, ferulate, coumarate, 1,2,4-benzene triol, 3- and 4-hydroxybenzoate, and catechin) that decreased in response to all treatments. Salicylate (2-hydroxybenzoate) also increased moderately in all stresses. In the water limitation treatment, multiple aromatic conjugates (purpurin and five partially identified caffeoyl conjugate peaks) increased. Copper treatment and water limitation showed similar responses in the phenolic glycosides, with increases in nearly all of the metabolites detected in this group. Overall, the shade-treated plants responded in the opposite direction of copper and water limitation treatments with respect to the phenolic glycoside group. Water and light limitation resulted in similar expression patterns of sugar and sugar acids, though the changes were more pronounced in light-limited plants. In light-limited plants, fructose decreased from 8,800 µg/g of fresh weight (gFW) in controls to only 835 µg/gFW. Raffinose and sugar alcohols (including arabitol, ribitol, glycerol, and *myo*-inositol) increased in water-limited plants. Both erythronate and threonate were decreased in response to all treatments, and other conjugated sugars decreased in shade plants, consistent with decreased metabolism in leaves. We next correlated gene expression data with metabolite concentrations (Pearson *R*, Data Set S1). Of the 1,000 most significant correlations, azelaic acid was the most highly correlated with gene expression data (60 genes), followed by the amino acids lysine (35 genes) and serine (28 genes). In *Arabidopsis*, azelaic acid is a signaling factor in systemic resistance that affect salicylic acid signaling and resistance to *P. syringae* pathogens (57).

### Microbiome changes in response to stress.

Microbiome community composition in the root and rhizosphere was measured by 16S amplicon sequencing and, at the phylum level, is similar to that observed in other poplar microbiome studies, with a high abundance of *Proteobacteria* and representation of *Actinobacteria* and *Verrucomicrobia* (25, 26). Primary analyses of root (including both the internal and external root environments) and rhizosphere communities showed clustering on the basis of isolation compartment, and thus, further analyses were performed separately (Fig. S1; Data Set S1).

**FIG 4:**
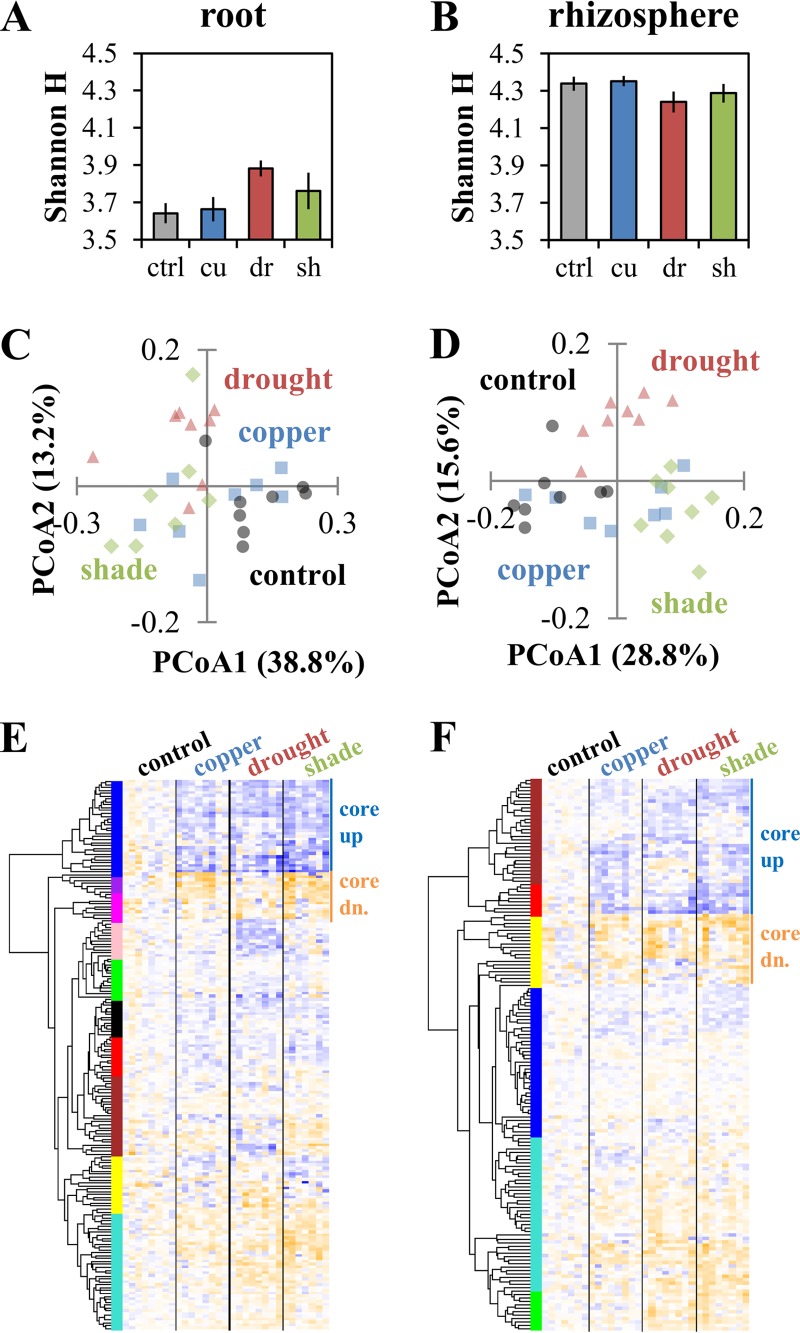
Belowground microbiome community responses. (A) Shannon diversity index (H) for OTUs with >0.01% relative abundance in the root compartment. (B) Shannon diversity index (H) for OTUs with >0.01% relative abundance in the rhizosphere compartment (ctrl, control; cu, copper; dr, drought; sh, shade). (C) Weighted UniFrac PCoA of root communities showing clustering of communities by condition. (D) Weighted UniFrac PCoA of rhizosphere communities showing clustering of communities by condition. (E) Bacterial OTUs upregulated (blue) or downregulated (dn.; orange) in response to stress in the root microbiome, with distance-based clusters identified by using the hclust and dynamic tree cut packages in R. (F) Bacterial OTUs up- or downregulated in response to stress in the rhizosphere, with distance-based clusters identified by using the hclust and dynamic tree cut functions in R.

Bacterial diversity, as measured by the Shannon H index, increased in root communities under water limitation and shade treatment but decreased in rhizosphere communities under the same treatments (Fig. 4A and B). Microbial communities were compared by using a weighted UniFrac distance metric and clustered by treatment (Adonis, *P* < 0.001) for both root and rhizosphere communities (Fig. 4C and D; part 1of Data Set S1). Analysis of clusters showed that treatments resulted in significant shifts in communities relative to the control and that treatments also resulted in different communities relative to each other (Table 1). Clustering analysis of the community data (Fig. 4E and F) indicated that a cluster of operational taxonomic units (OTUs) either increase or decrease in abundance in response to treatment, with directionality and magnitude similar between treatments.

**TABLE 1:** Statistical tests of treatments^a^

Community and treatment	Copper	Drought	Shade
Root			
Control	5.3 × 10^−3^,^b^ 5.1 × 10^−1^	2.8 × 10^−6^,^c^ 5.5 × 10^−3^^b^	1.4 × 10^−10^,^c^ 4.4 × 10^−2^^d^
Copper		7.2 × 10^−2^, 2.7 × 10^−3^^b^	5.5 × 10^−2^, 6.9 × 10^−1^
Drought			1.4 × 10^−3^,^b^ 7.4 × 10^−1^
Rhizosphere			
Control	1.0 × 10^−2^,^d^ 1.3 × 10^−1^	5.2 × 10^−7^,^c^ 3.2 × 10^−8^^c^	1.7 × 10^−10^,^c^ 1.1 × 10^−11^^c^
Copper		1.3 × 10^−3^,^b^ 4.2 × 10^−7^^c^	3.4 × 10^−1^, 1.7 × 10^−2^d
Drought			4.9 × 10^−7^,^c^ 5.5 × 10^−7^^c^

a Results of 999 Monte Carlo permutations to determine the significance of differences between weighted UniFrac distance metrics for communities subjected to different treatments. The first value is the result of a pairwise test of distance within a column treatment compared to the distance between column and row treatments. The second value is for the distance within a row treatment compared to the distance between row and column treatments.

b *P* < 0.01.

c *P* < 0.001.

d *P* < 0.05.

**FIG 5:**
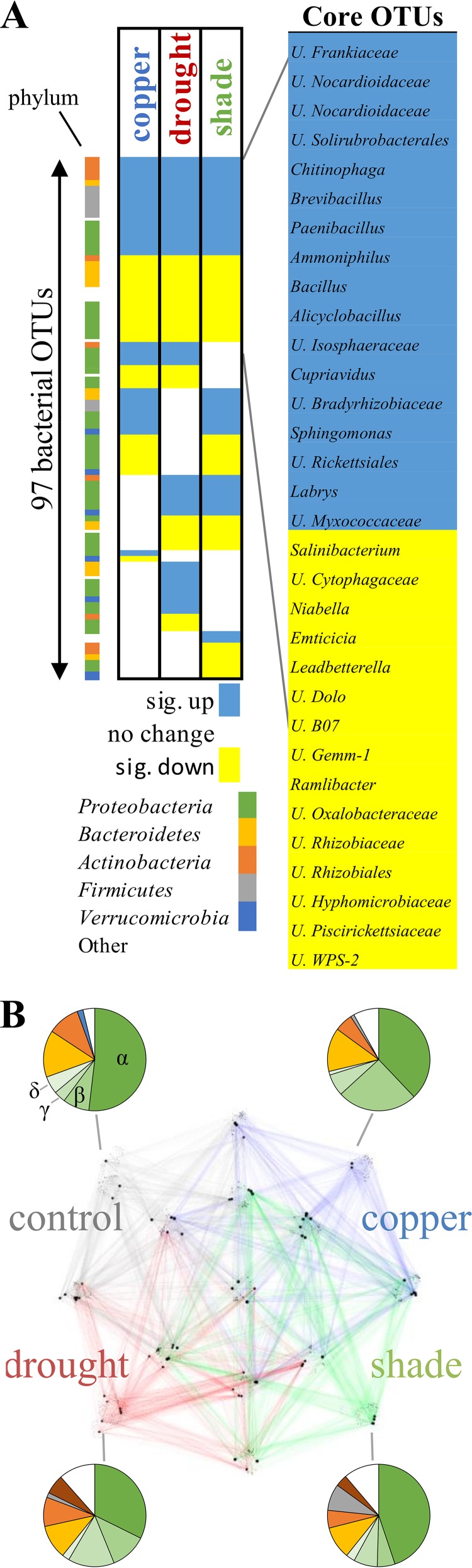
Summary of the core genera in the root microbiome and their correlation structure. (A) OTUs were identified as significantly (sig.) up- or downregulated by using Student's *t* tests with FDR correction (α = 0.1). Significantly increased OTUs are indicated by yellow fill, decreased OTUs are indicated by blue fill, and no change is indicated by white fill. On the right are the genus level identifications of core OTUs (U stands for unidentified, to distinguish OTUs classified to higher taxonomic levels). Phyla are mapped to colors as shown by the key in panel A, with green shades representing *Alphaproteobacteria*, *Betaproteobacteria*, *Gammaproteobacteria*, or *Deltaproteobacteria* as shown and white segments corresponding to unlisted phyla. (B) Correlation network analysis indicates individual treatment networks and OTUs correlated under multiple treatments. Pie charts indicate the taxonomy of nodes in subnetworks.

We next investigated which specific root OTUs changed in response to each stress condition. By this approach, we found 97 OTUs representing *Proteobacteria*, *Bacteroidetes*, *Actinobacteria*, *Firmicutes*, and *Verrucomicrobia* (representing 10.3 to 14.9% relative abundance) significantly increased or decreased (Student's *t* test with false-discovery rate [FDR] correction, *P* < 0.05, with α = 0.10) in at least one treatment (Fig. 5A; Data Set S1). Of the 97 OTUs that were significantly increased or decreased in abundance, 68 were significant in more than one treatment, with the direction of change consistent in every case. We observed similar behavior in rhizosphere data, with only one example of an OTU having an opposite directional change (Data Set S1). Overall, OTUs representing 10 to 14% of the total increased or decreased in all stress treatments, while additional OTUs representing 0.3 to 7% of the total increased or decreased in abundance in response to specific treatments only (Table 2).

**TABLE 2:** Distribution of stress-responsive OTUs

No. of treatments/total	Relative abundance as % of total community (no. of OTUs)		
	Copper	Drought	Shade
3/3	10 (32)	13 (32)	14 (32)
2/3	12 (23)	16 (21)	18 (28)
1/3	0.3 (2)	7 (12)	0.5 (8)

To determine the predictability of stress responses in rhizosphere and root microbial communities, we built a naive Bayes classifier using OTUs (present in at least 80% of the samples) as features and treatments as labels. Prediction accuracy was tested by using a leave-one-out strategy (Data Set S1). In the root microbial community, the classifier predicted 21/31 treatments correctly. Within the stress treatments, the classifier predicted 21/23 cases as stress conditions (gray box in part 3 of Data Set S1). Further, control samples were correctly predicted eight of eight times, with only two instances of stress-treated samples predicted as controls. Analysis of the rhizosphere community showed similar results, with 19/24 stress treatments predicted correctly as stress. Control and copper samples overlapped, with two controls predicted as copper treatments and four copper treatments predicted as controls. Interestingly, one of the drought-treated plants (plant J) was predicted as a control in both root and rhizosphere analyses.

To study microbial community structure, we calculated correlations between taxa within the microbiome community by using SparCC (58). The top 10,000 edges were used to generate networks for each treatment, and then the 20 most connected nodes from each treatment and their immediate neighbors were selected to visualize subnetworks for each treatment (Fig. S4). The subnetworks from each treatment were then merged to determine OTUs with correlations unique to treatments or shared between treatments (Fig. 5B). Of the 807 OTUs included in the merged network, 108 were only correlated with others in the control treatment, with 87, 130, and 107 in the three stresses (copper, drought, and shade). These treatment-specific subnetworks had similar taxonomic profiles dominated by *Proteobacteria*, *Actinobacteria*, and *Bacteroidetes* OTUs. There were 15 OTUs correlated with OTUs in all treatments (center subnetwork), including seven *Proteobacteria* and four *Bacteroidetes* OTUs (Data Set S1). Bacteria within these OTUs may be important in shaping the overall structure of the poplar root microbiome.

10.1128/mSystems.00070-17.3 FIG S3  Elemental analyses of leaf tissues. Elemental composition was determined by X-ray fluorescence. An additional 11 metals (Ca, Fe, Ni, Pd, Rh, Si, Ti, Va, and Zn) were tested and were not significantly changed (*, *P* > 0.05 [Student's *t* test]). Error bars are 1 standard error from four independent samples. Download FIG S3, TIF file, 0.5 MB. Copyright © 2018 Timm et al. 2018 Timm et al. This content is distributed under the terms of the Creative Commons Attribution 4.0 International license.

10.1128/mSystems.00070-17.4 FIG S4  Co-occurrence correlation network for identification of cutoff. (A) Number of edges at increased correlation cutoffs (coefficients determined by SparCC). (B) Pairwise correlation of overall node ranks. (C to K) Pairwise correlation plots of overall node ranks when ordered by numbers of edges (*x* versus *y*). Download FIG S4, TIF file, 2.6 MB. Copyright © 2018 Timm et al. 2018 Timm et al. This content is distributed under the terms of the Creative Commons Attribution 4.0 International license.

The 97 OTUs identified as increased or decreased in abundance, which we term the “core stress OTUs,” were analyzed for correlation with gene expression data or metabolite concentrations (Pearson correlation with a *P* value cutoff of <0.05; Data Set S1). Genes that showed the highest number of correlations with OTUs included a transmembrane protein kinase (*Populus trichocarpa* 013G030100, *Arabidopsis thaliana* homolog At1g057000) that was previously identified in a stress response study (59). OTUs with the highest number of correlations included an OTU identified in the A4b family of the *Anaerolineae* class which have little described function in ecosystems but are hypothesized to contribute to carbohydrate degradation in anaerobic digesters (60). Other uncultured OTUs included the NKB19 and WPS-2 phyla. OTUs within the *Rhizobiales* order and the *Acidobacteriaceae* family were also identified as more frequently correlated with gene expression data (Data Set S1). Core stress OTUs were correlated (positively and negatively) with multiple metabolites in the phenolic glycoside and sugar conjugate groups. OTUs correlated with multiple phenolic glycosides (2-*O*-salicyloylsalicin, salicortin, salicin, and grandidentatin) or sugar conjugates (purpurein caffeoyl-3-*O*-quinic acid) include *Brevibacillus*, *Oxalobacteraceae*, *Paenibacillus*, and *Niabella*, which all have plant growth-promoting representatives. Within the drought-responsive group, we observed OTU correlations between amino acid metabolites and aromatic metabolites. *Burkholderia*, *Verrucomicrobia*, *Xanthomonadaceae*, and *Acidobacteria* OTUs show correlations with lysine and glutamine, catechol, and caffeoyl-shikimate conjugates. Finally, in shade-responsive OTUs, we observed correlations in the phenolic glycoside, sugar metabolism, and sugar conjugate groups, consistent with significant changes in these metabolite groups in shade-treated plants. *Aeromicrobium*, *Spirosoma*, and *Luteolibacter* OTUs and an unidentified *Betaproteobacteria* OTU correlate with multiple metabolites in these groups.

## DISCUSSION

In both natural and agronomic ecosystems, poor growth conditions can limit plant productivity and ultimately decrease the biomass yield. In this work, we present a systems level approach to the study of the phytobiome response to environmental treatments that induce plant stress. Using a belowground microbiome inoculation study and functional measures of plant growth, including gas exchange and fluorescence, plant transcriptional response, metabolite profile response, and microbiome community response, we show that the plant and associated belowground bacteria exhibit both stress-specific and core stress responses. This study suggests that the core microbiome members identified above appear to be tightly coupled to the physiology of the host plant and highlights the need for further testing to identify mechanisms of community change and consequences for phytobiome function and fitness.

Plant responses, as measured by growth patterns, gas exchange and productivity, and leaf gene and metabolite expression profiles, indicated that plants were stressed in response to metal toxicity, water, and light limitation. However, the severity of the stress was likely different among copper, drought, and shade treatments. Consistent with previous studies, we observed decreases in plant productivity, shifts in gene expression toward the production of cell wall components, and decreases in photosynthetic processes (61) in water-limited plants. Previous work has shown that stress severity in drought treatments impacts the plant response at the physiological and gene expression levels (62), which presumably has subsequent downstream effects on the microbiome of the plant. The drought treatment in this study was cyclic, acute, and implemented in accordance with the individual plant response. With this approach, some plants experienced two drought cycles and others as many as five throughout the treatment period; however, a consistent response in terms of a microbiome effect was observed (Fig. 4 and 5). There may be a differential response in plants maintained under long-term low-water conditions. Drought conditions affect both plant and soil environments, and these soil environmental effects likely contributed to the observed changes in the microbiome, especially in the rhizosphere (63). In contrast, the light limitation treatment is more specifically a host effect, limiting changes in the soil environment compared to water limitation. However, the reduced water requirements of shaded plants, as well as the decreased soil temperature owing to lack of direct sunlight may have affected environmental conditions and indirectly contributed to changes in the microbiome. In this work, copper stress was likely the least severe of the three treatments, as indicated by growth measurements and transcriptional responses. The lower stress severity may explain the observed weaker changes in the microbiome response. Conversely, the antimicrobial properties of copper may also have affected root-associated microbes directly or indirectly (37), which might contribute to the significant response in the gene expression results, which showed increases in the biotic stress pathway. Despite the diverse environmental changes imposed by these treatments, we did observe a common response in the microbiome community structure (Fig. 4 and 5) that is best explained by the influence of the stressed host plant. The observation of a core stress microbiome was further supported by the results from a naive Bayes classifier in which stress samples that were incorrectly identified were primarily identified as one of the complementary stress treatments (Data Set S1).

The metabolic environment of the host contributes to the structure of the microbiome, either by modifying the metabolites available and the resulting competition or by direct inhibition of specific microbes. In water-limited plants, we observed consistent changes in metabolite profiles as consistent with other studies, which show increases in amino acids, phenolic compounds, and soluble sugars and sugar derivatives in leaves (64–67). Similarly, we observed changes in shaded plant metabolite profiles consistent with other studies of light limitation (32, 33, 68). In Scots pine (*Pinus sylvestris*) trees, shading leads to lipid-dominated respiration, as opposed to the carbohydrate-dominated respiration that is observed in water-limited trees (69). In *Stellaria*, shading changed the composition of gibberellins and auxin (70). Both shade and drought conditions have been shown to modulate carbohydrate, amino acid, and lipid contents in *Pinus* trees (69). However, it is not possible to conclude from this study or past studies whether changes in leaf metabolite profiles are a direct response to the environment and lead to changes in the microbiome or if the changes are a feedback result of changes in the microbial community. Plant metabolites have been shown to impact the microbial community (55), and inoculation by root microbes has been shown to impact leaf metabolite profiles (17), confounding the cause-effect relationship between the plant and its microbiome. Correlation analyses between OTUs and metabolites suggest that phenolic glycosides or other sugar conjugates may be driving plant-microbe interactions in this system, supporting the hypothesis that the plant host controls microbiome community members through differential feeding or inhibition of competitors. In addition, the identification of azelaic acid as highly correlated with gene expression data suggests some level of systemic resistance response to stresses (57), potentially contributing to the microbes associated with the plant. *Anaerolineae*, uncultured phyla, *Rhizobiales*, and *Acidobacteriaceae* OTUs were correlated with gene expression data, indicating a potential relationship between the microbiome response and the plant response (Data Set S1). Further work is needed to elucidate the relationships among gene expression, metabolite production, and OTU abundances in order to understand and predict microbiome interactions with host plants.

In this work, we studied the endpoint response of the belowground microbiome to plant stress. The analyses performed here and additional studies may enable strategies for controlling the microbiome to achieve reduced stress in plants. Of great future interest will be the dynamic response of the phytobiome to environmental stressors to determine both the time scale of functional responses and the implications for the microbial community associated with the plant. We did measure the photosynthesis kinetics of drought-treated plants and observed a functional response at the phytobiome level, but it is unclear how the microbiome responds during this dynamic time in the environment. Some microbes may be fast responders, on the order of hours, while others respond on the order of weeks. Additionally, the recovery of the plant microbiome as a community after host stress is unknown. Understanding how the microbiome rebounds after stress will also help us identity which microbes are important contributors to phytobiome function. Ultimately, it is the hope of the phytobiome community that we will be able to use this to harness the adaptive power of the microbiome and predictably modulate the system response.

Our bacterial community results indicate that high-level taxonomy may be indicative of microbiome structure, with detailed functional changes attributed to specific OTUs. Despite changes in relative abundances and correlation structures, we did observe that high-level taxonomy (phylum and order) was similar between treatments and similar to other poplar (25, 26) and other plant microbiome (71) studies. Uniqueness thus appears at lower taxonomic levels (family and below). This pattern may be associated with the broad phylogenetic relationship of complex phenotypes in the *Bacteria* kingdom. While some unique bacterial phenotypes are distributed within a phylum, complex phenotypes tend to be conserved at the family level or a higher level (72). Therefore, there is likely some commonality in the stressed environment or community that imposes the observed distribution of phyla in plant microbiomes. Further analyses identifying mechanisms leading to the observed stress response in the microbiome are required.

In this work, we showed the response of the plant-microbiome system to diverse environmental conditions. Ideally, these results will inform future studies to generate and modulate communities with predictable and beneficial effects on the host plant.

## MATERIALS AND METHODS

### Germfree plants.

*P. deltoides* WV94 clones were maintained in greenhouses at Oak Ridge National Laboratory (Oak Ridge, TN) with 16-h days supplemented with 1,000-W high-pressure sodium halide lamps. Shoot tips collected from actively growing plants were sterilized by washing in 10% bleach, 70% ethanol, and five times in deionized water. Tips were rooted in tissue culture medium (1× Murashige and Skoog basal salt mixture, 5 g/liter charcoal, 30 g/liter sucrose, 1 ml/liter plant preservative mixture) to produce rooted cuttings. Initial rooted cuttings were serially cultured in the same medium to generate germfree experimental plants.

### Microbiome isolation.

The natural microbiome used for inoculation in the microbiome inoculation study was collected by harvesting 20 g of fine roots from a *P. deltoides* tree in the Oak Ridge National Laboratory complex in September 2014. Roots were washed with sterile water, ground in 10 mM MgSO_4_ with a mortar and pestle, and then centrifuged at 10,000 × *g* for 10 min to pellet the root-associated microbes. The pellet was resuspended in 25% glycerol and then stored at −80ºC until inoculation. After freezing, a sample was thawed to determine the number of CFU per milliliter to use for subsequent inoculation calculations. Axenic rooted cuttings of *P. deltoides* WV94 were planted in double-autoclaved soil inoculated with a natural microbiome isolated from wild *P. deltoides*.

### Greenhouse conditions and treatments.

*P. deltoides* WV94 rooted cuttings were subcultured and rerooted in fresh medium. Rooted cuttings were selected and planted in 150 ml of autoclaved potting mix (Farfard 4M) mixed with 100 ml of microbiome inoculum at ~10^6^ CFU/ml suspended in sterile Hoagland’s No. 2 Basal Salt Mixture (Caisson Laboratories) (32 plants for stress study) or 100 ml of sterile Hoagland’s No. 2 Basal Salt Mixture (8 plants for uninoculated controls). After inoculation, plants were acclimated to greenhouse conditions via growth chamber (12 days) and then greenhouse (17 days at a photosynthetically active radiation [PAR] level of 500 µmol m^−2^ s^−1^), and plant stress treatments (eight plants per treatment) were initiated when plants reached a height of ~20 cm. Control plants were watered every day with Southern Ag 20/10/20 at 100 ppm dissolved in MilliQ-treated water. Cyclic water limitation stress was implemented on the basis of the individual plant response; specifically, plants were watered when the first fully expanded leaf drooped with the main vein parallel to the primary stem. Shade cloth that blocked 90% of the incoming radiation was used for light limitation, and shaded plants were watered as needed (about every 2 to 3 days). Copper sulfate (30 µM) was added to nutrient solution to achieve a final concentration of 34 µM to induce metal toxicity stress, and plants were watered daily. Treatments were applied for 22 days.

Plants were acclimated to the greenhouse, and the stress experiment was performed between 23 October and 14 November 2014 with a day/night cycle of 16/8 h. Natural light was supplemented with 1,000-W high-pressure sodium halide lamps. Control plants were watered to capacity every day with a 100 ppm Southern Ag nutrient solution. Water limitation stress was applied in cycles on the basis of the response of each plant. Plants were watered to capacity at extreme wilting (first full leaf vein parallel to stem). Plants were watered with the same nutrient solution with added 30 µM CuSO_4_ (pH balanced to nutrient solution, pH 5.34). Shade-treated plants were grown under 90% shade, leading to a maximum PAR level of 80 µmol m^−2^ s^−1^. Shaded plants were watered as needed, approximately every 3 days.

### Plant growth and physiology.

Once every 7 days, all plants (eight per condition) were measured for chlorophyll content, shoot height, leaf number (leaves longer than 2 cm), and branch count (any branch containing a leaf longer than 2 cm). Chlorophyll content was measured on the fourth, fifth, and sixth fully expanded leaves with a SPAD-502Plus (Konica Minolta, Ramsey, NJ). Shoot height was measured from the base of the stem to the highest actively growing leaf. Leaves were counted beginning at the first leaf >2 cm long. When the experiment was concluded, all leaves were scanned to measure the total leaf area and leaf samples were collected and measured for average LMA (mg/cm^2^) by using three 1-cm leaf punches per plant taken from mature leaves, with statistical tests comparing the eight plants per treatment.

Gas exchange and chlorophyll fluorescence measurements were taken with an open photosynthesis system (LI6400XT; LI-COR, Lincoln, NE) fitted with a chlorophyll fluorescence chamber (6400-40; LI-COR Inc.). On days 12 to 18, gas exchange and chlorophyll fluorescence were measured once daily on two representative plants from the water limitation and control treatments at dark-adapted and PAR levels of 400 and 2,000 µmol m^−2^ s^−1^. On days 15 and 16, gas exchange and chlorophyll fluorescence were measured in four additional plants from the copper, water limitation, shade, and control treatments.

The first fully expanded leaf of each plant measured was selected on day 12; gas exchange and fluorescence measurements were taken on this same leaf throughout the experiment. Before beginning the photosynthesis measurements, the leaves were dark adapted for 30 min. Each measurement included one dark-adapted fluorescence measurement that was combined with a gas exchange measurement. The dark-adapted measurement was followed by gas exchange measurements at ambient and maximum light levels. The ambient and maximum photosynthetic photon flux densities (PPFDs) were measured at 400 and 2,000 µmol m^−2^ s^−1^, respectively. Chamber conditions were kept at a constant CO_2_ flow rate of 400 ppm, and the relative humidity was controlled at 60 to 70%. Before measurement, the dark-adapted leaf was given 2 to 3 min to stabilize inside the chamber. For PPFD measurements at both 400 and 2,000 µmol m^−2^ s^−1^, samples were allowed to stabilize for 5 ± 1 min inside the chamber.

### RNA sequencing and analysis.

Stored leaf tissue was ground in liquid nitrogen, and total RNA was extracted by combining a cetyltrimethylammonium bromide (CTAB) lysis buffer method and a Spectrum plant total RNA extraction kit (Sigma-Aldrich, St. Louis, MO). Approximately 100 mg of flash-frozen ground tissue was incubated in 850 μl of CTAB buffer (1.0% β-mercaptoethanol) at 56°C for 5 min, 600 μl of chloroform-isoamyl alcohol (24:1) was added, and samples were centrifuged at 14,000 × *g* for 8 min. The supernatant was removed and applied to the Spectrum plant total RNA extraction kit filter column (Sigma-Aldrich, St. Louis, MO). RNA was precipitated in 500 μl of 100% ethanol and applied to the Spectrum plant total RNA extraction kit binding column, and subsequent washes and elution were completed in accordance with the manufacturer’s instructions, including the optional on-column DNase treatment to rid the samples of residual genomic DNA. RNA quality and quantity were determined with a NanoDrop 1000 spectrophotometer (Thermo Scientific, Waltham, MA) and a Qubit fluorometer (Thermo Scientific, Waltham, MA).

Total RNA (1 μg) was sequenced at Oak Ridge National Laboratory by using a single lane of an Illumina MiSeq (Illumina Inc., San Diego, CA) per plant for three biological replicates. Data handling and processing were performed on the basis of our pipeline (73). The raw reads were first evaluated for quality with SolexaQA++ toolkits (74). The high-quality reads (phred quality score, >25; length after trimming, >25 bases) were obtained with the BWA dynamic trimming algorithm in the SolexaQA++ toolkits, aligned with the *P. trichocarpa* v3.0 genome with bowtie2 (75), and then used to generate read counts for statistical analysis. The count tables were normalized for statistical analysis as proposed by Law et al. (76). The MapMan software (77) was used for analysis and statistical testing for pathway differential expression (*P* < 0.05, Wilcoxon rank sum test, Benjamini-Hochberg correction). Gene expression was further investigated by qRT-PCR analysis of a panel of *Populus* genes (Data Set S1). A RevertAid first-strand cDNA synthesis kit (Thermo Scientific, Waltham, MA) was used to synthesize cDNA from 3 μg of total RNA for subsequent qRT-PCR analysis. qRT-PCRs for plant targets were done by using SYBR green with ROX (Bio-Rad, Hercules, CA) in accordance with the manufacturer’s instructions, and reactions were run on an Applied Biosystems 7900HT instrument (Applied Biosystems, Foster City, CA).

### Metabolomics and elemental analyses.

Bulk leaf tissue was collected, flash frozen, and ground in liquid nitrogen, and then 50 µg was twice extracted overnight with 2.5 ml of 80% ethanol in water at room temperature. Sorbitol was added (to achieve a 10-ng/μl final concentration) before extraction as an internal standard to correct for differences in extraction efficiency, subsequent differences in derivatization efficiency, and changes in sample volume during heating. Extracts were pooled, and 1 ml of the extract was dried with a nitrogen stream. Dried extracts were dissolved in acetonitrile, *N*-methyl-*N*-trimethylsilyltrifluoroacetamide with 1% trimethylchlorosilane was added, and samples were then heated for 1 h at 70°C to generate trimethylsilyl (TMS) derivatives (78, 79). After 2 days, aliquots were injected into an Agilent 5975C inert XL gas chromatograph-mass spectrometer (Agilent, Santa Clara, CA). The standard quadrupole gas chromatograph-mass spectrometer is operated in the electron impact (70 eV) ionization mode, targeting 2.5 full-spectrum (50 to 650 Da) scans per second, as described previously (79). Metabolite peaks were extracted by using a key selected ion, characteristic *m/z* fragment, rather than the total ion chromatogram, to minimize the integration of coeluting metabolites. The extracted peaks of known metabolites were scaled to the total ion current by using predetermined scaling factors. Peaks were quantified by area integration, and the concentrations were normalized to the quantity of the internal standard recovered and the amount of sample extracted, derivatized, and injected. A large user-created database (~2,300 spectra) of mass spectral electron impact ionization fragmentation patterns of TMS-derivatized compounds, as well as the Wiley Registry 10th edition combined with the National Institute of Standards and Technology 2014 mass spectral library, was used to identify the metabolites of interest to be quantified. There were four replicate plants per treatment, and treatment differences were tested for statistical significance with Student's *t* tests. Data are presented as log_2_ fold changes, which were calculated by determining fold changes defined as absolute values of changes up or down and then scaled by taking the logarithm of the data and applying a plus or minus sign to indicate an increase or decrease in expression, respectively. Flash-frozen leaf tissues were ground in liquid nitrogen and then dried. Samples were analyzed in triplicate for 60 s with the Bruker Tracer III-SD X-ray fluorescence instrument (Bruker, Billerica, MA) and the included vacuum pump at a voltage of 15 kV and a current of 25 µA. Spectra were collected with the S1PXRF software and analyzed with the ARTAX software provided by Bruker. Data were exported and further analyzed with Microsoft Excel (Data Set S1).

### Bacterial community analysis.

Roots were collected from plants after 21 days of treatment. For 16S rRNA gene community analysis, the rhizosphere fraction was prepared by vortexing ~50 mg of roots in water and then pelleting the wash at 14,000 × *g* for 5 min. DNA was extracted from the pellet with the Mo Bio PowerSoil kit (Mo Bio Laboratories, Inc., Carlsbad, CA) in accordance with the manufacturer’s instructions. DNA was extracted from the remaining root material (here, the root) by homogenizing root tissue with three rounds of LN2 freeze and 1 min of bead beating, followed by the Mo Bio PowerPlant kit (Mo Bio Laboratories, Inc., Carlsbad, CA) in accordance with the manufacturer’s instructions.

The bacterial 16S rRNA gene was selectively amplified and barcoded by using established protocols utilizing PNA blockers to prevent plastid and mitochondrial 16S rRNA gene amplification (80). For initial primer ligation and amplification, the KAPA 2G PCR system was used with 515 forward and 806 reverse staggered primers for five PCR cycles. Following initial amplification, samples were bead purified (Agencourt AMPure XP) and then amplified with barcoded primers with the KAPA HiGi PCR system for 32 cycles. A total of 83 samples (root and rhizosphere for 40 plants plus three replicates of the inoculum) were pooled and then sequenced at Oak Ridge National Laboratory with a single 2 × 300 paired-end sequencing kit on Illumina MiSeq (Illumina Inc., San Diego, CA) with Nextera P1 primer. Reads were joined with the QIIME join_paired_ends script (81) by using default settings, unjoined reads were discarded, and then assembled reads were assigned to samples from barcodes by using split_libraries. Primers were removed with cutadapt (82) with a maximum error rate of 10%. OTUs were identified by open reference OTU picking by using the GreenGenes 13_5 97% database (83). Diversity analyses were run with the QIIME core_diversity_analysis script by using default parameters. Read counts ranged from 126,618 to 440,696 for root samples and 20,492 to 351,940 for rhizosphere samples, with one sample (root K) failing to sequence. Plant OTUs defined as reads clustering with mitochondrial chloroplast sequences were removed with QIIME (filter_taxa_from_otu_table). Samples were analyzed with the QIIME core_diversity_analysis script and rarefied to 19,000 reads to accommodate samples with the lowest read counts for combined analysis of root and rhizosphere communities. The resulting OTU table was analyzed with the weighted UniFrac distance metric (84) by using principal-coordinate analysis (PCoA) and clustered by root or rhizosphere (Adonis, *P* < 0.001), and the data were thus subsequently analyzed separately with QIIME, R (dynamicTreeCut) (85), and Microsoft Excel as described in Data Set S1. For Shannon diversity calculations, OTUs present at >0.01% in samples were included to reduce noise associated with low-abundance taxa, and diversity was calculated with the formula *H* = Σ*p*_*i ⋅ *_ln(*p*_*i*_), where *p*_*i*_ represents the normalized population fraction of species *i*. Shannon diversity was averaged for eight plants per condition and compared in Student's *t* tests. A naive Bayes classifier was built with the python sklearn package by assuming a Gaussian distribution. OTUs present in at least 80% of the samples were use as features with treatments used as labels. For predictions, a leave-one-out strategy was implemented in which each sample was omitted from the training set and classified.

### Community structure analysis.

Co-occurrence correlation networks were determined for root samples by the SparCC method (58) for each stress condition. To capture OTUs consistently associated with plants in our experiment, OTU tables were filtered before network generation by omitting any OTU that occurred in <80% of the samples as a cutoff for potential contaminants and spurious reads. The average correlation out of 20 iterations was calculated, and then edges were selected to be significant at *P* values of <0.1 on the basis of 100 resampled OTU data sets, resulting in >100,000 edges per network. Network edges were reduced first by determining the relationship between the numbers of edges and nodes (Fig. S4A and B) and then by stepwise correlation (Fig. S4C to H). By this method, we selected the top 10,000 edges (sorted by descending correlation score) for analysis.

### Accession number(s).

Raw data obtained in this study were deposited in the Sequence Read Archive (SRA) database under accession number SRS1879507. Amplicon data obtained in this study are available in the SRA database under accession number PRJNA400863.
