# Supplementary figures and images for: Abiotic Stresses Shift Belowground Populus-Associated Bacteria Toward a Core Stress Microbiome
Source: mSystems. 2018 Jan 23;3(1):e00070-17. doi: 10.1128/mSystems.00070-17 (PMC5781258; doi:10.1128/mSystems.00070-17)

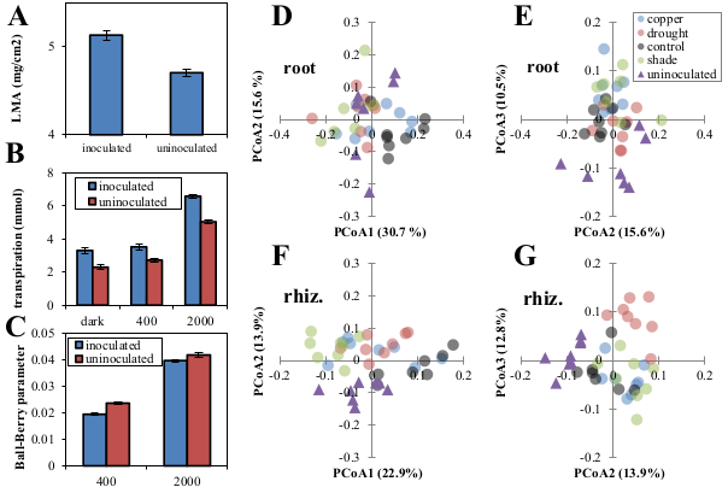

Supplement: FIG S1 [file sys001182164sf1.tif]

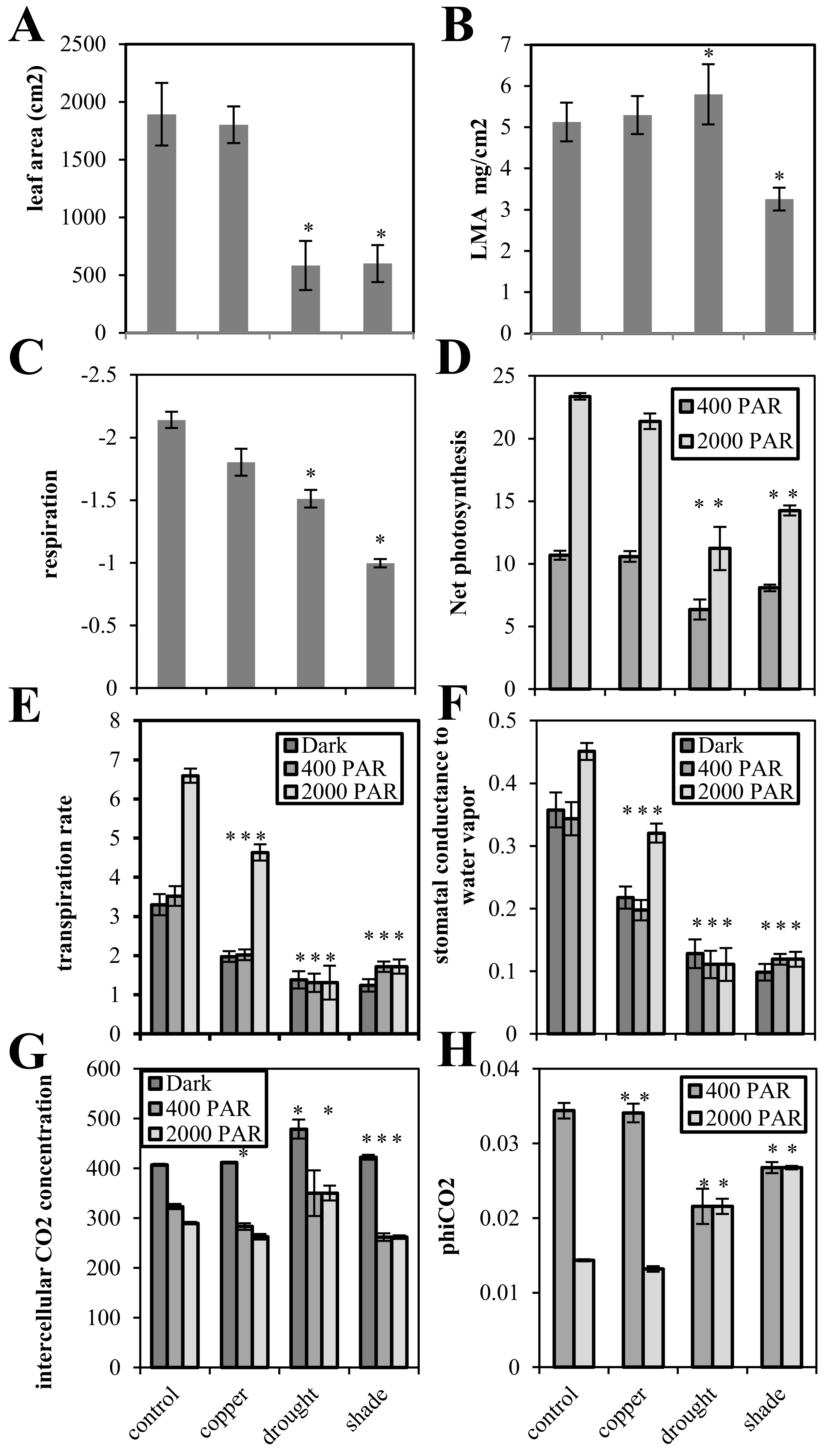

Supplement: FIG S2 [file sys001182164sf2.tif]

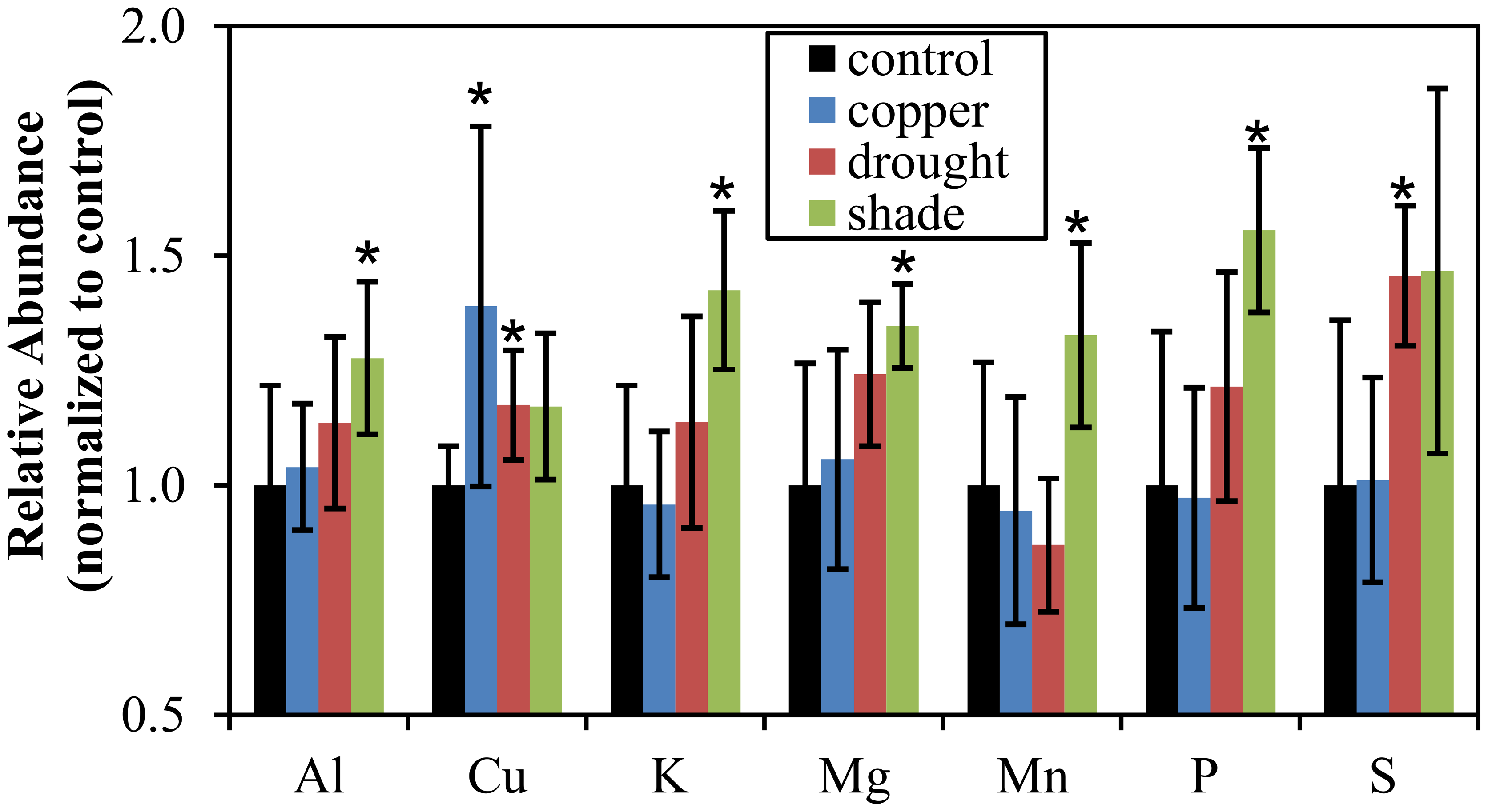

Supplement: FIG S3 [file sys001182164sf3.tif]

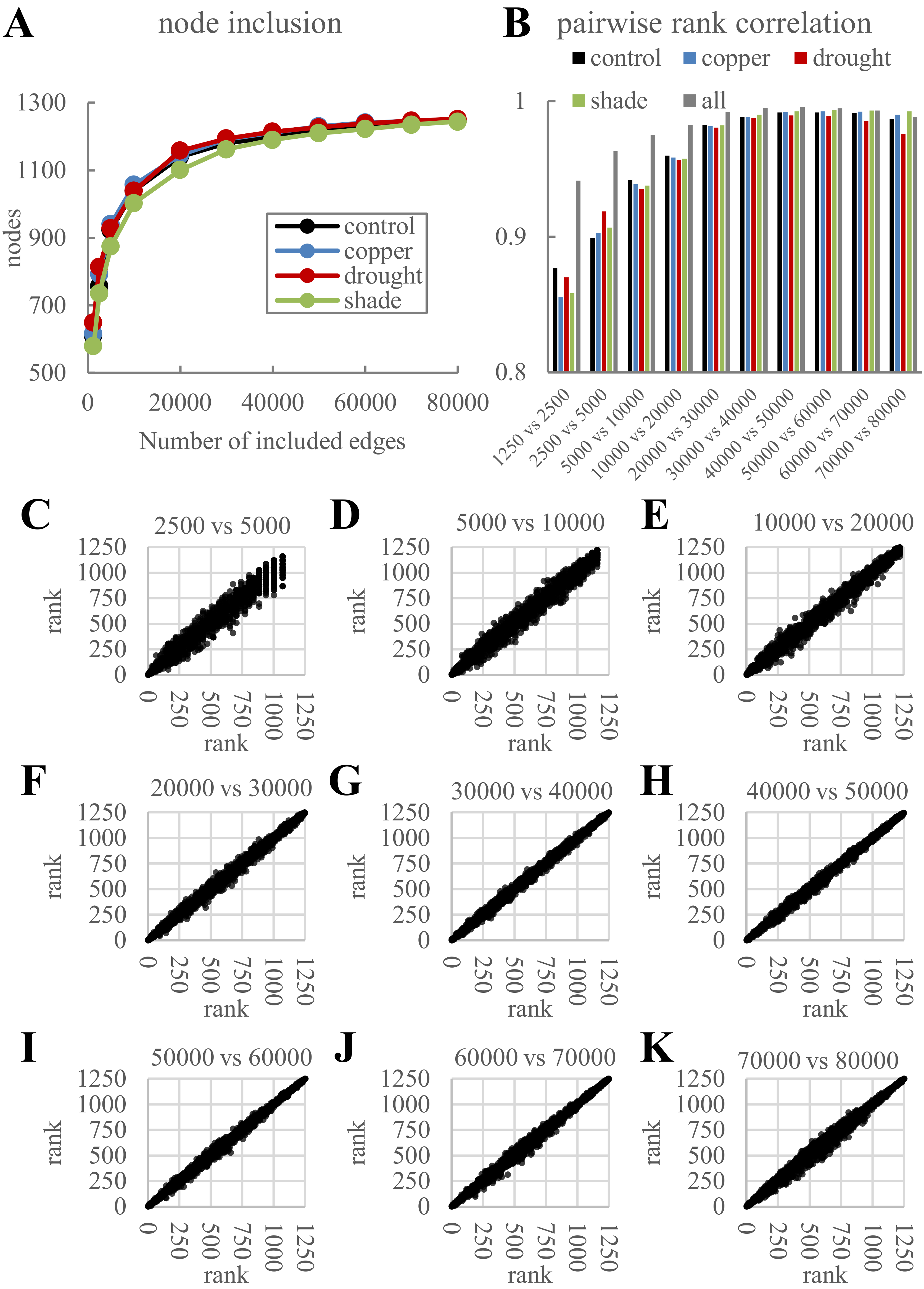

Supplement: FIG S4 [file sys001182164sf4.tif]
